# Patient safety culture in Norwegian nursing homes

**DOI:** 10.1186/s12913-017-2387-9

**Published:** 2017-06-20

**Authors:** Gunnar Tschudi Bondevik, Dag Hofoss, Bettina Sandgathe Husebø, Ellen Catharina Tveter Deilkås

**Affiliations:** 1 Research Group for General Practice, Department of Global Public Health and Primary Care, University of Bergen, Bergen, Norway; 2 National Centre for Emergency Primary Health Care, Uni Research Health, Bergen, Norway; 3 Institute of Health and Society, University of Oslo, Oslo, Norway; 4 Centre for Elderly and Nursing Home Medicine, Department of Global Public Health and Primary Care, University of Bergen, Bergen, Norway; 5 Municipality of Bergen, Bergen, Norway; 6 The Norwegian Directorate of Health, Oslo, Norway; 7 Health Services Research Unit, Akershus University Hospital, Lørenskog, Norway

**Keywords:** Adverse events, Medical errors, Norway, Nursing homes, Patient safety culture, Quality improvement, Safety attitudes questionnaire

## Abstract

**Background:**

Patient safety culture concerns leader and staff interaction, attitudes, routines, awareness and practices that impinge on the risk of patient-adverse events. Due to their complex multiple diseases, nursing home patients are at particularly high risk of adverse events. Studies have found an association between patient safety culture and the risk of adverse events. This study aimed to investigate safety attitudes among healthcare providers in Norwegian nursing homes, using the Safety Attitudes Questionnaire – Ambulatory Version (SAQ-AV). We studied whether variations in safety attitudes were related to professional background, age, work experience and mother tongue.

**Methods:**

In February 2016, 463 healthcare providers working in five nursing homes in Tønsberg, Norway, were invited to answer the SAQ-AV, translated and adapted to the Norwegian nursing home setting. Previous validation of the Norwegian SAQ-AV for nursing homes identified five patient safety factors: teamwork climate, safety climate, job satisfaction, working conditions and stress recognition. SPSS v.22 was used for statistical analysis, which included estimations of mean values, standard deviations and multiple linear regressions. *P*-values <0.05 were considered to be significant.

**Results:**

Out of the 463 employees invited, 288 (62.2%) answered the questionnaire. Response rates varied between 56.9% and 72.2% across the five nursing homes. In multiple linear regression analysis, we found that increasing age and job position among the healthcare providers were associated with significantly increased mean scores for the patient safety factors teamwork climate, safety climate, job satisfaction and working conditions. Not being a Norwegian native speaker was associated with a significantly higher mean score for job satisfaction and a significantly lower mean score for stress recognition. Neither professional background nor work experience were significantly associated with mean scores for any patient safety factor.

**Conclusions:**

Patient safety factor scores in nursing homes were poorer than previously found in Norwegian general practices, but similar to findings in out-of-hours primary care clinics. Patient safety culture assessment may help nursing home leaders to initiate targeted quality improvement interventions. Further research should investigate associations between patient safety culture and the occurrence of adverse events in nursing homes.

## Background

Patient safety, and how to learn from medical errors, is attracting increasing attention, both in hospital and primary care [1–7]. Patient safety culture is leadership and staff interaction, attitudes, routines, awareness and practices that impinge on the risk of patient-adverse events [8]. The concept is regarded as a group, rather than an individual, phenomenon. Safety culture is most important in the health service units closest to the patients, such as wards [9]. Variation in safety culture is associated with the risk of adverse events [10–12].

Nursing home patients are at high risk of adverse events. These individuals are particularly vulnerable due to their age, cognitive impairment, complex multiple diseases, and non-specific presentation of illnesses [13]. There is also an increased risk of fall injuries and errors due to multiple medication and potential drug interactions [14, 15]. Studies by Soraas et al. (2014) and Gulla et al. (2016) found that the use of eight or more drugs is frequent in Norwegian nursing homes, leading to increased risk of drug-drug interactions, which in turn may lead to falls, cognitive decline, medication-related problems, and even increased mortality [16–20]. In nursing homes, physicians frequently prescribe drugs without a proper clinical evaluation of the patient, and staff may not be aware of this [21].

Furthermore, it is widely recognised that the assessment and treatment of acute and persistent pain in nursing home patients and people with dementia is a complex entity, with reported prevalence of up to 60% in this population [22]. Meanwhile, almost one half of the Norwegian population dies in a nursing home, so that end-of-life care and advance care planning addressing the approaching death, and the practical challenges regarding ethics, treatment and care, should be initiated well before the patient reaches a critical state [23].

In Norwegian nursing homes there is a high ratio of employees with limited training. Medical physicians are often only present for one day per week. To ensure that treatment and care are in accordance with the wishes of the patient and the family, highly motivated and competent healthcare personnel is required.

Patient safety culture in nursing homes has been found to be poorer than in hospitals [24–26]. Studies have found an association between patient safety culture in nursing homes and clinical outcomes, such as the prevalence of patients injured due to falls [27, 28].

Clinical settings dominated by a “blame and shame” culture may face challenges in implementing safety improvement projects [29–31]. Patient safety culture assessments give leaders the opportunity to address cultural obstacles – and also to set quality improvement targets [8, 29].

A number of instruments have been developed to measure safety attitudes among healthcare providers. The two most widely used are the Hospital Survey on Patient Safety Culture (HSOPSC) and the Safety Attitudes Questionnaire (SAQ) [32, 33]. Based on the original versions, these questionnaires have been adapted to different settings in primary care, including the Nursing Home Survey on Patient Safety Culture (NHSOPSC) [26, 34], and the Safety Attitudes Questionnaire – Ambulatory Version (SAQ-AV) [1]. Both instruments have been validated for Norwegian nursing homes [35] ﻿(Bondevik GT, Hofoss D, Husebø BS, Deilkås ECT: ﻿The safety attitudes questionnaire – ambulatory version: psychometric properties of the Norwegian version for nursing homes, submitted). The validation of the SAQ-AV for nursing homes in Norway identified five major patient safety factors: teamwork climate, safety climate, job satisfaction, working conditions and stress recognition. The SAQ has proved to be a useful tool to measure safety culture in nursing homes in other countries [36, 37]. SAQ scores correlate with patient outcomes, such as surgical site infections, decubitus ulcers and other adverse events [10–12, 38–41]. The instrument may identify weaknesses in patient care and motivate quality improvement interventions, leading to a reduction of the risk of adverse events [42–44].

Besides validation for the nursing home setting, the Norwegian version of the SAQ-AV has been validated for out-of-hours (OOH) clinics and regular general practitioner (GP) practices [45, 46]. This allows for comparison of patient safety cultures across different areas of the healthcare services. Patient safety culture scores vary considerably across work sites in primary care (Deilkås ECT, Hofoss D, Hansen EH, Bondevik GT: Variation in staff perceptions of patient safety climate between Norwegian General Practitioner (GP) practice and Out-of-hours clinic work sites, submitted).

In this paper, we studied whether variations in safety attitudes in Norwegian nursing homes, as measured by the SAQ-AV, were related to professional background, age, work experience and mother tongue amongst the healthcare providers.

## Methods

This study was conducted in all five nursing homes in Tønsberg, a Norwegian town with 42,000 inhabitants. The number of patients in each nursing home varied between 38 and 101, and in total there were 366 patients. The total number of employees in the nursing homes was 765. In order to investigate the safety culture in the nursing home wards, we excluded healthcare providers with a job position less than/equal to 20%, or those who were on leave during the study period (*n* = 302). Most of these were employees working only one weekend every third or fourth week. The remaining 463 employees invited worked more than 20% in the nursing homes (Table 1), and were mostly registered nurses and nursing assistants.

**Table 1:** Patients, employees and response rates in five nursing homes in Tønsberg, Norway, 2016

	Patients (n)	Employees total (n)	Employees ≤20% & leave (n)	Employees >20% invited (n)	Respondents (n)	Response rate (%)
Nursing home 1	46	80	26	54	39	72.2
Nursing home 2	38	65	17	48	29	60.4
Nursing home 3	92	201	51	150	95	63.3
Nursing home 4	101	215	92	123	70	56.9
Nursing home 5	89	204	116	88	55	62.5
Total	366	765	302	463	288	62.2

Employees ≤20% & leave = Number of employees having a job position ≤20% in a nursing home, or on leave during the study period

Employees >20% invited = Number of invited employees having a job position >20% in a nursing home

### Translation procedures

The original SAQ-AV questionnaire was translated in accordance with modified principles adapted from Beaton et al. [47]. Initially, the original English version was translated into Norwegian by a professional translation agency. Then, an expert committee with clinicians and researchers from the Research Group for General Practice (University of Bergen) and the Health Services Research Unit (Akershus University Hospital) adapted the initial translated version to the primary care setting in Norway. This adapted version of the questionnaire was translated back into English by a second independent translation agency, and was blinded to the original version. Based on this back-translated version, an expert committee made adjustments in order to clarify possible misunderstandings and adapt the questionnaire to the Norwegian nursing home setting. For instance, the original SAQ-AV statement “Medical errors are handled appropriately in this office” was changed to “Medical errors are handled appropriately in this nursing home ward”, and “Nurse input is well received in this office” was changed to “Staff input is well received in this nursing home ward”. The pre-final version was evaluated by a group of healthcare providers in nursing homes. Based on their feedback, the final version of the Norwegian translated questionnaire was developed. Pre-tests showed that it took approximately 15 min to complete the SAQ-AV.

### Scoring

The SAQ-AV is a 62-item questionnaire whereby the respondents rate their agreement using a 5-point Likert scale: 1 = disagree strongly, 2 = disagree slightly, 3 = neutral, 4 = agree slightly, 5 = agree strongly. For all questions, “Not applicable” was included as a response category, and combined with missing values in the data analyses. Scores of negatively worded items were reversed, so that higher scores in the data set always indicate a more positive evaluation of the patient safety culture in the nursing home. Mean scores for the patient safety factors were calculated using the following formula: (mean scores of items belonging to the factor – 1) × 25 [41]. The validation of the Norwegian SAQ-AV for nursing homes identified five patient safety factors: teamwork climate, safety climate, job satisfaction, working conditions and stress recognition (Table 2). The quality of collaboration and communication with relevant professional groups and services was rated on the following scale: very low, low, adequate, high and very high.

**Table 2:** Patient safety factors and corresponding items, based on 169^a^ respondents in five nursing homes, Norway. (Bondevik GT, Hofoss D, Husebø BS, Deilkås ECT: ﻿The safety attitudes questionnaire – ambulatory version: psychometric properties of the Norwegian version for nursing homes, submitted)

Teamwork	Input from personnel is well received in this nursing home ward.
climate	In this nursing home ward, it is difficult to speak up if I perceive a problem with patient care^b^.
	Disagreements in this nursing home ward are resolved appropriately (i.e., not *who* is right, but *what* is best for the patient). I have the support I need from other personnel to care for patients.
Safety	I would feel safe being treated here as a patient.
climate	Medical errors are handled appropriately in this nursing home ward.
	I receive appropriate feedback about my performance.
	In this nursing home ward, it is difficult to discuss errors^b^.
	I am encouraged by my colleagues to report any patient safety concerns I may have.
	The culture in this nursing home ward makes it easy to learn from the errors of others.
	I know the proper channels to direct questions regarding patient safety in this nursing home ward.
Job	I like my job.
satisfaction	Working in this nursing home ward is like being part of a large family.
	This nursing home ward is a good place to work.
	I am proud to work at this nursing home ward.
	Morale in this nursing home ward is high.
Working	This nursing home ward does a good job of training new personnel.
conditions	All the necessary information for diagnostic and therapeutic decisions is routinely available to me.
	The levels of staffing in this nursing home ward are sufficient to handle the number of patients.
	This nursing home ward deals constructively with problem personnel.
	Trainees in my discipline are adequately supervised.
Stress	Briefing other personnel before a procedure (e.g. wound care) is important for patient safety.
recognition	When my workload becomes excessive, my performance is impaired.
	I am less effective at work when fatigued.
	I am more likely to make errors in tense or hostile situations.
	Stress from personal problems adversely affects my performance.

^a^169 of the healthcare providers included in this study replied to each of the items (they had no missing/not applicable for any of the items related to the five factors)

^b^Reverse-scored items

### Data collection

Data were collected in February 2016. Information about the study was presented on posters in the nursing home wards and in handouts to all participants prior to – and during – data collection. The administrative keypersons in the nursing homes distributed a paper version of the SAQ-AV to their employees, and reminded them one week before the deadline. To ensure confidentiality, completed questionnaires were returned anonymously in boxes placed in the nursing home wards. The questionnaires were scanned optically into SPSS files.

Each of the participating nursing home wards received a report showing the SAQ-AV results for their own ward compared anonymously with all other participating wards, so that no random reader could identify which other wards were described in the report. The healthcare providers were encouraged to focus on specific factors related to patient safety in their own ward, and to discuss possible improvement strategies. In order to protect the confidentiality of the respondents, reports were only produced for wards with five respondents or more.

### Statistical analysis

IBM SPSS Statistics for Windows, Version 22.0. Armonk, NY: IBM Corp. was used for statistical analysis, which included estimation of mean values, standard deviations and multiple linear regressions. *p*-values <0.05 were considered significant.

### Ethical considerations

This study was based on data regarding patient safety culture among healthcare providers in nursing homes. It was conducted in compliance with the ethical guidelines of the Helsinki Declaration. All participants received written information about the purpose of the study, and were assured that the data would be collected anonymously and treated in confidence. The study was approved by the Norwegian Social Science Data Services – the governmental agency for protecting survey research respondent privacy in accordance with the Norwegian Personal Data Act (Ref. No. 2015/42892). As the study was not affected by the Norwegian Health Research Act, approval from an ethics committee was not required.

## Results

Out of the 463 healthcare providers invited, working more than 20% in the nursing homes, 288 (62.2%) responded to the questionnaire. Response rates varied between 56.9% and 72.2% across the five nursing homes (Table 1). Among those responding to the questionnaire, the average proportion of items with missing values/not applicable was 9.4%.

The basic characteristics of the participating subjects are presented in Table 3. Three quarters of the respondents were either registered nurses or nursing assistants, and the vast majority were female. Approximately 40% of the participants were above 50 years of age, and one half had total work experience exceeding 20 years. One third of the employees had been working at the nursing home for more than ten years. Most of the participants worked on a part-time basis. Nearly one fifth were not native Norwegian speakers.

**Table 3:** Characteristics of 288 employees in five nursing homes, Norway, responding to the SAQ-AV, 2016

		Number	Percent
Profession	Registered nurse	78	30
	Nursing assistant	124	47
	Health worker	41	16
	Kitchen personnel	7	3
	Laundry personnel	3	1
	Secretary	1	0.4
	Other personnel	9	3
	Missing	25	
Gender	Female	241	94
	Male	16	6
	Missing	31	
Age	≤ 30 years	47	18
	31–40 years	44	17
	41–50 years	65	25
	51–60 years	78	30
	≥ 61 years	30	11
	Missing	24	
Work experience	≤ 5 years	44	17
in total	6–10 years	29	11
	11–20 years	60	23
	21–30 years	68	26
	31–40 years	56	21
	≥ 41 years	9	3
	Missing	22	
Work experience	≤ 2 years	59	22
in nursing home	3–5 years	60	22
	6–10 years	56	21
	11–20 years	57	21
	21–30 years	30	11
	≥ 31 years	6	2
	Missing	20	
Position job	21–40%	30	12
	41–60%	42	16
	61–80%	78	30
	≥ 81%	107	42
	Missing	31	
Norwegian native	Yes	226	83
speaker	No	47	17
	Missing	15	

Proportions (%) not including missing data

Table 4 describes the quality of collaboration and communication experienced by the study participants with regard to relevant professional groups and services. Almost 90% of the participants described the quality of collaboration and communication with registered nurses and nursing assistants as “high” or “very high”. The corresponding ratio for physicians was 71%, while the lowest percentage with “high” and “very high” scores was observed for collaboration and communication with nursing home management (56%), physiotherapists/occupational therapists (51%) and the municipal nursing home admission authority (42%).

**Table 4:** Described quality of collaboration and communication among 288 employees in five nursing homes, Norway, 2016

Quality of collaboration and communication with^a^	Very low n (%)	Low n (%)	Adequate n (%)	High n (%)	Very High n (%)	N/A^b^ n
Physicians	5 (3)	13 (7)	40 (20)	62 (31)	81 (40)	54
Registered nurses	1 (0.4)	4 (2)	26 (10)	97 (39)	123 (49)	6
Nursing assistants	2 (1)	2 (1)	25 (10)	91 (36)	135 (53)	4
Health workers	2 (1)	3 (1)	39 (16)	109 (43)	98 (39)	5
Kitchen personnel	2(1)	12 (5)	56 (24)	88 (38)	73 (32)	24
Cleaning personnel	7 (3)	27 (12)	48 (21)	87 (37)	64 (28)	23
Laundry personnel	5 (3)	9 (5)	37 (19)	76 (38)	73 (37)	51
Administrative secretaries	2 (1)	9 (4)	51 (23)	85 (38)	77 (34)	25
Physio/Occupational therapists	15 (10)	23 (16)	35 (24)	35 (24)	40 (27)	98
Nursing home management	9 (4)	20 (8)	79 (32)	85 (35)	51 (21)	5
Municipal authority^c^	5 (4)	15 (13)	45 (40)	34 (30)	14 (12)	129
Out-of-hours service	3 (2)	10 (7)	34 (25)	61 (45)	28 (21)	110
Ambulance staff	4 (3)	7 (5)	36 (24)	67 (45)	34 (23)	97
Hospital	3 (2)	4 (3)	41 (29)	60 (43)	32 (23)	103

^a^The study participants responded to the following question: “With respect to your experiences at this nursing home, use the scale to describe the quality of collaboration and communication that you have experienced with the following groups:”

^b^Not Applicable. Proportions (%) in the table not including participants responding “Not Applicable”

^c^Municipal nursing home admission authority

Total mean scores for the five identified patient safety factors, and corresponding mean scores by profession, age, position job, work experience and mother tongue are presented in Table 5. *P*-values were obtained from the multiple linear regression model, adjusted for the variables included in the table.

**Table 5:** Patient safety factor scores by characteristics among employees in five nursing homes, Norway, 2016

		Teamwork climate			Safety climate			Job satisfaction			Working conditions			Stress recognition	
	n	mean (SD)	p	n	mean (SD)	p	n	mean (SD)	p	n	mean (SD)	p	n	mean (SD)	p
Total	254	72.5 (19.4)		221	70.8 (18.0)		257	81.3 (17.9)		217	69.9 (17.9)		239	73.9 (19.8)	
Profession^a^			0.39			0.37			0.16			0.80			0.63
Reg. nurse	73	75.0 (17.8)		70	70.6 (17.9)		70	80.4 (18.4)		67	72.7 (14.7)		69	78.0 (18.0)	
Nursing assist.	116	75.5 (18.4)		103	73.1 (17.0)		113	83.0 (15.0)		100	69.5 (18.5)		115	73.7 (21.2)	
Health worker	35	66.4 (16.9)		26	69.1 (18.2)		38	80.3 (17.2)		28	65.2 (21.7)		34	68.8 (16.2)	
Age			**0.03**			**0.02**			**0.00**			**0.00**			0.07
≤ 30 years	43	65.4 (21.6)		34	64.8 (18.0)		44	74.6 (20.3)		36	60.6 (16.9)		38	70.5 (18.6)	
31–40 years	41	73.8 (15.8)		36	69.5 (15.3)		41	78.8 (19.0)		34	68.1 (16.4)		38	69.7 (23.2)	
41–50 years	56	73.1 (18.2)		52	69.1 (19.0)		58	81.4 (17.1)		51	70.5 (17.3)		55	75.8 (20.4)	
51–60 years	70	75.9 (17.7)		61	74.1 (17.6)		70	85.6 (13.4)		61	73.9 (18.9)		67	75.7 (19.0)	
≥ 61 years	28	78.1 (18.8)		23	78.4 (14.6)		26	89.6 (13.1)		20	76.5 (13.4)		25	77.6 (15.7)	
Position job			**0.00**			**0.01**			**0.02**			**0.00**			0.08
21–40%	26	68.3 (17.4)		19	65.6 (22.4)		26	79.8 (19.6)		19	63.4 (22.9)		24	67.3 (22.2)	
41–60%	38	67.6 (16.2)		32	65.6 (15.7)		41	77.1 (14.1)		34	61.9 (17.4)		37	72.6 (18.9)	
61–80%	73	73.6 (19.4)		64	72.1 (17.7)		74	81.8 (17.4)		64	72.0 (17.9)		69	75.5 (19.7)	
≥ 81%	92	76.5 (19.7)		85	73.9 (17.3)		93	83.6 (18.6)		81	74.0 (14.9)		86	76.7 (19.2)	
Work experience in nursing home^b^			0.11			0.12			0.18			0.81			0.06
≤ 2 years	51	71.3 (17.8)		41	67.4 (18.0)		53	78.7 (19.0)		40	68.9 (15.9)		46	77.1 (18.4)	
3–5 years	55	66.7 (21.0)		51	66.3 (19.5)		55	76.9 (21.4)		49	62.8 (18.2)		52	75.0 (20.1)	
6–10 years	51	76.1 (17.8)		39	75.4 (16.8)		51	82.4 (13.7)		41	73.1 (16.4)		47	71.2 (21.1)	
11–20 years	52	76.8 (17.0)		44	74.0 (13.8)		51	85.6 (15.5)		44	74.1 (14.8)		47	68.7 (21.0)	
21–30 years	25	74.8 (20.6)		25	74.4 (20.8)		27	84.8 (15.9)		24	71.3 (21.7)		26	77.3 (16.6)	
≥ 31 years	5	78.8 (24.5)		5	73.6 (12.8)		4	91.3 (4.8)		5	72.0 (28.0)		5	81.0 (8.9)	
Norwegian native speaker			0.11			0.08			**0.01**			0.26			**0.00**
Yes	200	72.5 (19.3)		177	70.6 (17.8)		201	80.6 (17.9)		173	70.1 (17.2)		186	76.6 (17.3)	
No	42	76.6 (17.7)		35	74.4 (18.3)		44	86.1 (15.6)		36	70.4 (21.1)		41	62.7 (26.6)	

*P*-values obtained from multiple linear regression model adjusted for the other four explanatory variables in the model. *P*-values <0.05 indicating statistical significance in bold

^a^Profession: not including other professions, such as staff working in the kitchen, laundry, secretary and other services (total *n* = 20)

^b^Number of years of work at the nursing home

Older respondents had significantly higher mean scores for the patient safety factors teamwork climate, safety climate, job satisfaction and working conditions. Mean scores for stress recognition also tended to increase with increasing age, but not significantly in the adjusted analysis (*p* = 0.07).

Higher job position was also associated with significantly higher mean scores for teamwork climate, safety climate, job satisfaction and working conditions. Mean scores for stress recognition tended to increase with increasing part-time presence at work, but this relationship was not significant in the multiple linear regression analysis (*p* = 0.08).

Not being a Norwegian native speaker was associated with a significantly higher mean score for job satisfaction. The mean score for stress recognition was significantly lower among participants that were not Norwegian native speakers. The same group tended to perceive the safety climate as being better, although not significantly (*p* = 0.08).

Neither profession nor work experience were significantly associated with mean scores for any patient safety factor.

## Discussion

Our study showed that the Norwegian nursing home employees considered their collaboration and communication with registered nurses and nursing assistants to be of high quality. Their collaboration and communication with nursing home management, physio/occupational therapists and the municipal nursing home admission authority received lower scores. Increasing age and higher job position were associated with statistically significantly increased scores for the patient safety factors teamwork climate, safety climate, job satisfaction and working conditions. Not being a Norwegian native speaker was associated with a significantly higher mean score for job satisfaction and a significant lower mean score for stress recognition.

Although suboptimal, the overall response rate of 62% in our study was acceptable, and higher than the 53% response rate in a Dutch nursing home study [36], and the 52% response rate in a corresponding SAQ study of Norwegian GP practices and OOH clinics [45]. Among those responding to the questionnaire, there were moderate instances of items with missing values/not applicable, on average 9.4%. The substantial number of employees in the nursing homes working in part-time positions may have reflected the degree of attachment to the workplace, and thereby possibly the interest in participating in the study. Another weakness may be the difficulties that employees who are not Norwegian native speakers may have experienced with understanding the statements in the questionnaire. It is a strength of the present study that it was performed in all five nursing homes in an average-sized Norwegian town, including both small (38 patients, 65 employees) and large (101 patients, 215 employees) nursing homes. Introducing the commonly used SAQ instrument in the nursing home setting also presents an opportunity to compare safety cultures across different parts of the healthcare services.

The launch of the Norwegian Coordination Reform (2008) “Proper treatment – at the right place and right time” (https://www.regjeringen.no/en/dokumenter/report.no.-47-to-the-storting-2008-2009/id567201/) and the Dementia Plan 2015 by the Department of Health encourages municipalities to develop services and staff competence to improve the mental health of people with dementia and reduce the need for specialist healthcare services [48]. Increased staff competence is a prerequisite for successfully meeting the new requirements arising as a consequence of the Coordination Reform. To date, care services are provided to more than 200,000 users in the primary healthcare system, of whom 41,000 live in one of the 900 nursing homes. In Norway, about 49% of the population die in a nursing home, 35% in a hospital, and only 15% at home [49]; which is the lowest proportion for home deaths, in worldwide terms. With limited resources, new solutions are necessary, including competence improvement in home care services and better collaboration with general practitioners, which is also related to nursing home services [50].

As three quarters of the respondents in our study were either registered nurses or nursing assistants, it may not be surprising that the quality of collaboration and communication with these two professions was experienced as particularly high: inter-colleague interaction was considered to be good. The lower scores for collaboration and communication with physicians might be due to the limited part-time presence of this group in nursing homes, since many physicians work mainly as general practitioners.

It is interesting that collaboration and communication with the higher hierarchical layers, such as the nursing home management and the municipal nursing home admission authority, were found wanting. This may be a key problem, as attitudes and decisions made by the nursing home management and the municipal nursing home admission authority are likely to have significant consequences for job satisfaction and working conditions among clinical staff in nursing homes. From a patient safety perspective, it is important that the collaboration and communication between administrative and clinical personnel are as good as possible. Our findings in Norwegian nursing homes indicate that there might be room for improvement.

The significant association between higher age/job position and higher scores for the patient safety factors teamwork climate, safety climate, job satisfaction and working conditions lends itself to different interpretations. This may well be related to a higher degree of attachment to the workplace among more experienced and career-minded employees. Older respondents and those who spend more time at work might feel more comfortable in the nursing home clinical setting. On the other hand, younger employees may be a group that identifies possible risks easier. They are more recently trained and have probably been introduced to the issue of patient safety to a wider degree than their older colleagues. This may have created increased patient safety awareness, which was reflected in the lower scores in this group – and may explain why several patient safety factors were perceived as being poorer among the younger employees.

Not being a Norwegian native speaker was associated with a significantly higher mean score for job satisfaction and a significantly lower mean score for stress recognition. The same group also tended to score higher for safety climate, but not significantly higher. The country of origin is not known for the 47 respondents in our study who were not Norwegian native speakers. Some of them may have come from other Scandinavian countries, with a reasonable understanding of the Norwegian language. Others may have had difficulties with understanding the statements in the Norwegian version of the SAQ-AV. Due to these uncertainties, we should be cautious about explaining the findings for non-native speakers. However, it is interesting that the reported level of stress recognition was substantially lower for this group. It can be questioned whether this finding may be related more to concerns regarding employment security, rather than expressing a relaxed attitude to stress situations in this group, compared to Norwegian native speakers. Recognition of stress is regarded as an important factor to reduce risk and protect patients from medical errors. The significantly higher score for job satisfaction may reflect how non-native speakers perceive their work, and that they are more satisfied than their colleagues. Once again, varying cultures, traditions and attitudes to elderly people could explain the difference. Respondents with job experience from systems with low employment security may be more likely to appreciate the benefits of having a job.

Three of the SAQ patient safety factors confirmed in the nursing home setting (safety climate, job satisfaction and working conditions) included exactly the same items as the confirmatory factor analyses in Norwegian GP practice and out-of-hours (OOH) services [45, 46]. This makes it possible to compare these three factor scores across the primary healthcare services. The findings from the present nursing home study showed a similar overall mean score for safety climate (70.8 ± 18.0) as for OOH (69.6 ± 18.1), but lower than for GP practices (77.2 ± 17.8). The corresponding figures for job satisfaction were (81.3 ± 17.9), (83.4 ± 16.1) and (87.6 ± 13.1), and for working conditions (69.9 ± 17.9), (69.2 ± 21.2) and (76.2 ± 18.1). This shows that patient safety factor scores in nursing homes are lower than in GP practices, but are comparable with findings in OOH clinics, another primary healthcare service with increased risk of adverse events [46]. Our findings are in accordance with the results from other studies where the safety culture in nursing homes has been found to be poorer than in other healthcare services [24–26, 29]. In the Netherlands, however, SAQ scores for nursing homes were either higher than or comparable with scores for other healthcare services – both hospital and ambulatory services [36]. As nursing home patients are regarded as particularly vulnerable individuals at great risk of adverse events, there is a need for improvement of the safety culture amongst healthcare providers.

It is important to emphasise, however, that high scores for patient safety factors in nursing home settings are not necessarily associated with a high quality of diagnostic skills, adequate treatment and patient-centred care. Employees who are satisfied with their own performance at work may tend to score high on the SAQ, perhaps also in situations where patient care is not optimal. For this reason, leaders of nursing homes obtaining high patient safety scores should interpret the results with caution – and still consider implementing quality improvement interventions. To explore this concern further, there is a need to investigate the associations between patient safety culture and the occurrence of adverse events in nursing homes.

## Conclusions

Our study showed that increasing age and job position were associated with higher scores for the patient safety factors teamwork climate, safety climate, job satisfaction and working conditions. Not being a Norwegian native speaker was associated with a higher mean score for job satisfaction and a lower mean score for stress recognition. Patient safety factor scores in nursing homes were poorer than in GP practices, but similar to findings in OOH clinics. SAQ results may help nursing home leaders to initiate targeted quality improvement interventions. These may include improving working conditions and job satisfaction, reducing the risk of medical errors related to drugs and fall injuries, and improving the quality of the communication among employees. In a forthcoming study we will explore nursing home ward-level variation in safety culture measurements. Further research should also validate the SAQ externally by correlating the safety factor scores to the prevalence of adverse events in nursing homes.

## Abbreviations

GP: General practitioner
HSOPSC: Hospital survey on patient safety culture
NHSOPSC: Nursing home survey on patient safety culture
OOH: Out-of-hours
SAQ: Safety attitudes questionnaire
SAQ-AV: Safety attitudes questionnaire – ambulatory version
SPSS: Statistical package for the social sciences
